# Terpenoids from *Zingiber officinale* (Ginger) Induce Apoptosis in Endometrial Cancer Cells through the Activation of p53

**DOI:** 10.1371/journal.pone.0053178

**Published:** 2012-12-31

**Authors:** Yang Liu, Rebecca J. Whelan, Bikash R. Pattnaik, Kai Ludwig, Enkateswar Subudhi, Helen Rowland, Nick Claussen, Noah Zucker, Shitanshu Uppal, David M. Kushner, Mildred Felder, Manish S. Patankar, Arvinder Kapur

**Affiliations:** 1 Department of Obstetrics and Gynecology, Shandong University, Qi Lu Hospital, Ji Nan, China; 2 Department of Obstetrics and Gynecology, University of Wisconsin-Madison, Madison, Wisconsin, United States of America; 3 Department of Chemistry and Biochemistry, Oberlin College, Oberlin, Ohio, United States of America; 4 Department of Pediatrics, University of Wisconsin-Madison, Madison, Wisconsin, United States of America; 5 Center for Biotechnology, Siksha O Anusandhan University, Bhubaneswar, India; King Faisal Specialist Hospital & Research Center, Saudi Arabia

## Abstract

Novel strategies are necessary to improve chemotherapy response in advanced and recurrent endometrial cancer. Here, we demonstrate that terpenoids present in the Steam Distilled Extract of Ginger (SDGE) are potent inhibitors of proliferation of endometrial cancer cells. SDGE, isolated from six different batches of ginger rhizomes, consistently inhibited proliferation of the endometrial cancer cell lines Ishikawa and ECC-1 at IC_50_ of 1.25 µg/ml. SDGE also enhanced the anti-proliferative effect of radiation and cisplatin. Decreased proliferation of Ishikawa and ECC-1 cells was a direct result of SDGE-induced apoptosis as demonstrated by FITC-Annexin V staining and expression of cleaved caspase 3. GC/MS analysis identified a total of 22 different terpenoid compounds in SDGE, with the isomers neral and geranial constituting 30–40%. Citral, a mixture of neral and geranial inhibited the proliferation of Ishikawa and ECC-1 cells at an IC_50_ 10 µM (2.3 µg/ml). Phenolic compounds such as gingerol and shogaol were not detected in SDGE and 6-gingerol was a weaker inhibitor of the proliferation of the endometrial cancer cells. SDGE was more effective in inducing cancer cell death than citral, suggesting that other terpenes present in SDGE were also contributing to endometrial cancer cell death. SDGE treatment resulted in a rapid and strong increase in intracellular calcium and a 20–40% decrease in the mitochondrial membrane potential. Ser-15 of p53 was phosphorylated after 15 min treatment of the cancer cells with SDGE. This increase in p53 was associated with 90% decrease in Bcl2 whereas no effect was observed on Bax. Inhibitor of p53, pifithrin-α, attenuated the anti-cancer effects of SDGE and apoptosis was also not observed in the p53^neg^ SKOV-3 cells. Our studies demonstrate that terpenoids from SDGE mediate apoptosis by activating p53 and should be therefore be investigated as agents for the treatment of endometrial cancer.

## Introduction

In the year 2011, approximately 8,010 women died due to endometrial cancer and nearly 47,130 patients were newly diagnosed with this cancer [1]. In about 70% of the women with a diagnosis of endometrial cancer, the disease is found localized to the uterine corpus and five year survival is as high as 85% [2]. Advanced and recurrent endometrial cancer patients, enrolled in several gynecologic oncology group (GOG) trials for agents including platinum, taxanes and anthracyclines, rarely have complete responses to therapy [3]–[9]. Combination regimens show higher response rates, but the progression free period with these therapies is relatively low (5–7 months) with higher morbidity and continued lack of cure [10]. These statistics highlight the need for the development of novel and effective chemopreventive and chemotherapeutic agents for endometrial cancer.

Naturally occurring dietary components provide an important source of bioactive compounds that can serve as both chemopreventive as well as chemotherapeutic agents against endometrial and other types of cancers [11]. Our lab is currently investigating the anti-cancer properties of compounds present in the rhizomes of ginger (*Zingiber officinale*). These studies are supported by previous investigations demonstrating that dry ginger powder or solvent extracts of ginger roots induce cell cycle arrest and apoptosis in skin, breast, prostate, colon, and ovarian cancer cells [12]–[17]. Topical application of the ethanolic extract of ginger decreased the incidence, size, and the number of DMBA/TPA induced tumors in SENCAR mice [18].

The majority of the previous studies have concluded that the bioactive components of the dry powder and solvent extract of ginger rhizomes responsible for the anti-cancer activities are the phenolic compounds 4-, 6-, 8- and 10- gingerols, paradol, and shogaol, a product formed after drying or heating of the roots [19], [20]. These phenolic compounds, and especially the gingerols exhibit anti-proliferative and anti-angiogenic properties as demonstrated by *in vitro* and *in vivo* studies in various cancer models [13], [14], [16], [21]–[25]. Human colorectal cancer cells when treated with 6-gingerol, inhibited cell proliferation by inducing G1 cell cycle arrest and apoptosis [26]. Gingerols exhibit these anti-cancer effects via multiple mechanisms, which include protein degradation as well as β-catenin, PKC delta, and GSK3 beta pathways [26]. Studies in the ovarian cancer model have demonstrated that 6-shogaol inhibits the secretion of VEGF by the cancer cells [16]. 6-gingerol induces apoptosis in the prostate cancer cell line LnCaP by increasing the expression of p53 and Bax and simultaneously decreasing the expression of Bcl-2 [16], [17], [21].

In addition to the powdered ginger and the solvent extraction, bioactive compounds can also be isolated by steam distillation of this rhizomes [27], [28]. To the best of our knowledge, only limited studies have been conducted to demonstrate the anti-cancer properties of the steam distilled extracts of ginger. Chemical analysis of the steam distilled extract of ginger indicates that the previously identified bioactive phenolic compounds are present at very low concentration in the steam distilled extracts of ginger [27]. In the current study we demonstrate that the steam distilled extracts of ginger are potent mediators of apoptosis in endometrial cancer cells. Our studies suggest that one of the major bioactive components of the steam distilled extract of ginger is citral (a mixture of two terpenoid isomers, neral and geranial). We demonstrate that treatment of the endometrial cancer cells with the steam distilled extract of ginger results in significant increase in intracellular calcium, decrease in the mitochondrial membrane potential, increase in the expression of caspase 3, phosphorylation of P53, and a significant decrease in the expression of Bcl-2. The observations outlined in our studies demonstrate that the steam distilled extract of ginger and its bioactive components have the potential to be developed as chemopreventive and chemotherapeutic agents for endometrial cancer.

## Materials and Methods

### Reagents and Cell Lines

Pifithrin-α was purchased from Sigma Life Science. DMEM (Dulbecco’s Modification of Eagle’s Medium), RPMI-1640, Hanks Balanced Salt Solution (HBSS), and Dulbecco’s Phosphate Buffered Saline (DPBS) were from Cellgro (Manassas, VA). DiOC6, Ionomycin and Indo 1-AM were purchased from Life Technologies (Grand Island, NY). SuperSignal West Dura Extended Duration Substrate RIPA buffer and Protease Inhibitor Cocktail were from ThermoFisher Scientific (Waltham, MA). Primary caspase-3 Rabbit antibody, Bcl-2 Rabbit antibody, Bax Rabbit antibody, Phospho-P53 Mouse antibody and β-actin Mouse antibody were purchased from Cell Signaling Technology (Beverly, MA). Peroxidase-conjugated AffiniPure Goat Anti-Rabbit IgG antibody and Peroxidase-conjugated AffiniPure Goat Anti-Mouse IgG antibody were from Jackson ImmunoResearch Laboratories (West Grove, PA). FITC-Annexin V Apoptosis Detection kit was purchased from BD Pharmingen (San Diego, CA). ECC-1 [29], [30] and Ishikawa [31] cells were a gift from Drs. Elaine Alarid and David Olive (Madison, WI), respectively. SKOV-3 cells were purchased from ATCC (Manasas, VA).

### Cell Culture

Ishikawa cells were grown in DMEM and ECC-1 and SKOV-3 cells were grown in RPMI media supplemented with 10% fetal bovine serum (FBS) and 1% penicillin/streptomycin antibiotics in a 5% CO_2_ incubator.

### Steam Distillation of Ginger Rhizomes

Ginger rhizomes were obtained from local vendors, cleaned with distilled water and cut into 0.5 cm pieces. Approximately 250–300 g of the cut ginger pieces were transferred to the 1000 ml round bottom flask of the Clevenger steam distillation apparatus. The ginger roots were submerged in 500 ml of deionized water (18 MOhm-cm) and steam distillation was carried out for 4–6 hours by heating the flask. The oil separating in the Clevenger apparatus was lighter than water and was separated by periodically draining the liquid accumulating in the separation tube of the unit. The oil was immediately aliquoted in microfuge tubes and frozen until used in assays. The density of the oil was calculated to be 0.87 g/ml and this measurement was used to calculate the concentration of the extract used to conduct the biological assays.

### Cell Proliferation Assays

Effect of steam distilled ginger extracts, citral, and 6-gingerol on the proliferation of the cancer cell lines was determined by the 3-(4,5-dimethythiazol-2-yl)-2,5-diphenyl tetrazolium bromide (MTT) uptake method [32], [33]. Briefly, the cancer cells were plated in 96-well plate at a density of 5000 cells/well in their respective medium. The cells were then treated with various concentrations of ginger extract (0.025 µg, 0.25 µg, 2.5 µg, 6.25 µg and 12.50 µg/ml) and incubated at 37°C in a 5% CO_2_ environment for 24 h, 48 h and 72 h. After the designated time period, 20 µl 3-(4,5-dimethythiazol-2-yl)-2,5-diphenyl tetrazolium bromide was added to each well and the plates were incubated at 37°C for additional 3 h. The formazan crystals formed in the wells were dissolved in 100 µl DMSO. The absorbance was measured at 570 nm using a Spectra MAX 190 (Molecular Devices, Sunnyvale, CA).

### Combined Treatment of Cancer Cells with SDGE and Radiation or Chemotherapy

MTT assays were conducted to determine if SDGE enhanced the anti-proliferation effect of radiation or chemotherapy in the endometrial cancer cells. Ishikawa or ECC-1 cells were plated in multiple 96 well plates (5×10^3^ cells/well) on day 1 of the experiment. After allowing the cells to stabilize, media or SDGE were added to the wells containing the endometrial cancer cells on Day 2. Cells in some of the wells were also treated with cisplatin (5 µM) while others were irradiated with a single dose of 4 Gy using a Cesium-137 radiator. Following these treatments, the cells were cultured for 72 h at 37°C in 5% CO_2_ environment. Effect of the treatment on proliferation of the endometrial cancer cells was determined by conducting the MTT assays as described above.

### Gas Chromatography-Mass Spectrometry of SDGE

Separation and identification of compounds in SDGE samples used a Shimadzu GC-17A gas chromatograph equipped with a QP-5000 quadrupole mass analyzer (Shimadzu Scientific Instruments, Columbia, MD). Prior to analysis, 20 µL of freshly defrosted SDGE was dissolved in 1000 µL of pentane. 1 µl of this dissolved extract was injected manually to the gas chromatograph using a 1∶50 inlet split ratio and helium as the carrier gas at a flow rate of 1.4-ml/min. The gas chromatograph contained a nonpolar RTX-5MS column (30 m length, 0.25 mm ID, 0.25 µm film thickness; Restek, Bellefonte, PA.) Column temperature was initially 70°C followed by a ramp at 4°C/min to 180°C. Electron ionization detection was in full-scan, positive ion mode over a mass-to-charge ratio (m/z) range of 41 to 300. Compounds were tentatively identified by searching a NIST library and by comparison of arithmetic retention indices to values reported by Adams [34].

### Measurement of Apoptosis by Flow Cytometry

Apoptosis was measured using the FITC-Annexin V Apoptosis Detection kit (BD Pharmingen, San Diego, CA). Briefly, 2×10^6^ cells were treated with 0.25 µg/ml ginger extract with or without 100 µM Pifithrin-α. After incubation at 37°C for 0–16 h, the cells were washed twice with cold PBS and resuspended in 1× binding buffer, (10 mM HEPES/NaOH, pH 7.4, 140 mM NaCl, 2.5 mM CaCl_2_)at a concentration of 1×10^6^ cells/ml. Then 1×10^5^ cells in 100 µl binding buffer, were transferred to 5 ml tubes and stained with 5 µl of FITC-Annexin V and 5 µl propidium iodide (PI). The cells were gently vortexed and incubated at room temperature for 15 min. After washing the cells with 1× binding buffer to remove the excess FITC-Annexin V and PI, the cells were analyzed on a FACSCalibur flow cytometer. The data were analyzed using FlowJo software.

#### Cell cycle assay

The endometrial cancer cells were treated with SDGE (250 ng/ml or 2.5 µg/ml) for 24, 48, and 72 h. Following treatment, the cells were harvested, washed with PBS and fixed in 75% ethanol, washed with PBS, and stained with propidium iodide. Flow cytometry was then performed to analyze the samples for both apoptosis and cell cycle status as described earlier [35].

### Western Blot Analysis

After treatment of the cells with the steam distilled extracts of ginger, the cancer cells were washed with ice cold phosphate buffered saline (PBS) and lysed with RIPA buffer (Pierce, Rockford, IL) containing a protease inhibitor cocktail (Thermo Scientific, Rockford, IL). The total amount of protein in the lysate was determined by using the BCA assay (Pierce). Cell lysates were loaded at 25 µg/well onto a 7.5 or 12% resolving polyacrylamide gel and separated by electrophoresis, after which, the proteins were transferred to PVDF membranes. The membranes were blocked with 5% milk in Tris buffered saline and probed with the appropriate primary antibodies. Horseradish peroxidase conjugated secondary antibodies and SuperSignal West Dura Extended Duration Substrate (Thermo Scientific, Rockford, IL) were used for detection of the proteins on the blots. The films were scanned using FLUORCHEM 890 and Image J software was used to quantify the intensities of the bands.

### Mitochondrial Membrane Potential Assay

The endometrial cancer cells were grown in T25 tissue culture flasks. Exponentially growing cells were treated with 0.025 µg/ml or 0.25 µg/ml of ginger extract for 24 hrs. The cells were then washed and harvested. 1×10^6^ cells were added to each flow tube from untreated, 0.025 µg/ml and 0.25 µg/ml ginger extract treated cells. The cells were treated with 40 nM DiOC6 at 37°C for 30 min. The cells were then washed, resuspended in 400 µl of PBS containing 2% FBS and analyzed by FACSCALIBUR flowcytometer to assess the mitochondrial membrane potential.The data were analyzed using FlowJo software.

### Calcium Flux Measurements

The Ishikawa cells in the log phase of growth were harvested using trypsin. The cells (1.2×10^7^) were washed three times and suspended in 1 ml of 0.5% bovine serum albumin (BSA) containing Hanks buffered saline that did not contain any divalent cations. The cells were loaded with Indo 1-AM (2 µM) in the presence of 4 mM probenecid for 30 min at 37°C in 5% CO_2_ environment. The cells were then washed and resuspended in Dulbecco’s phosphate buffered saline containing 0.5% BSA and 1 mM CaCl_2_ to a final concentration of 2×10^6^ cells/ml. The cells were filtered through a 35 micron membrane filter prior to flow cytometry on LSR-II cytometer. Cells were initially analyzed for 3 min to determine the baseline intracellular calcium concentration. SDGE (0.025, 0.25, or 2.5 µg/ml) or Ionomycin (1 µM used as a positive control) were subsequently added to the cells and the change in the Indo-1 fluorescence was determined by continuously streaming the cells through the flow cytometer for approximately 7 mins. The data obtained were analyzed using FlowJo software.

### Statistical Analysis

Statistical analysis was done using the GraphPad Prizm software. The threshold for statistical significance is a probability of 0.05. The data was analyzed using unpaired T-test.

## Results

### Isolation of Steam Distilled Extracts from Ginger Roots

Essential oils can be conveniently isolated from ginger roots by steam distillation in a modified Clevenger apparatus. We have been able to isolate approximately 300 mg of essential oils from 250 g of ginger roots obtained from local commercial vendors. A total of five batches of ginger rhizomes, each obtained from a separate commercial source, were used to isolate the essential oils by steam distillation. Two of these five batches of ginger were obtained from local vendors in Bhubaneshwar, India and the steam distillation was also carried out on-site. The remaining three batches of ginger were obtained from vendors in Wisconsin and steam distillation of ginger was carried out in the laboratory- at University of Wisconsin. The yield of the essential oil was comparable between all batches of ginger roots used in this study and the density of SDGE was determined to be approximately 0.87 g/L.

### Steam Distilled Ginger Extract is a Potent Inhibitor of Endometrial Cancer Cell Proliferation

**Figure 1:**
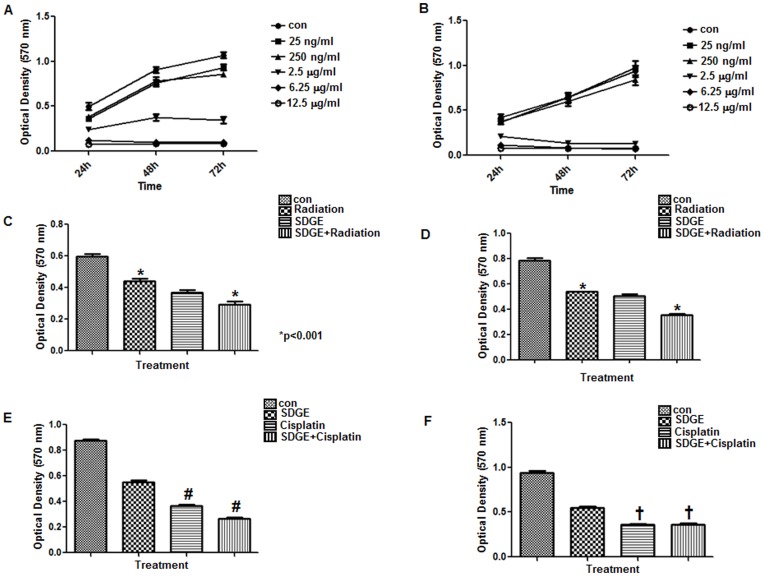
SDGE inhibits the proliferation of endometrial cancer cell lines. Effect of SDGE on cell proliferation was determined by conducting MTT assays. The cells Ishikawa (A), and ECC-1 (B) were incubated with the depicted concentrations of SDGE for 24, 48, and 72 h. Optical density at 570 nm was determined to quantify the number of live cells in the cultures. Control (Con) wells in all experiments were treated with DMSO, the vehicle control. Cumulative data for SDGE isolated from three different batches of ginger rhizomes is shown. Each data point in all figures is mean of 48 individual readings. Anti-proliferative effect produced by SDGE (2.5 µg/ml) in Ishikawa (C and E) and ECC-1 (D and F) cells was comparable to treatment of these two cell lines with radiation (C and D) or cisplatin (E and F). The cells were simultaneously treated with SDGE and radiation (4 Gy) or cisplatin (5 µM). MTT assays were conducted to determine the effect of these treatments on the proliferation of the Ishikawa and ECC-1 cell lines. Each bar in C–F represents a mean of eight replicates. *p<0.001, ^#^p<0.05, and ^†^p>0.05 (not significant).

The essential oils of ginger were first tested for their effect on the proliferation of two endometrial cancer (ECC-1 and Ishikawa) cell lines. Both endometrial cancer cell lines were sensitive to SDGE at concentration as low as 250 ng/ml (Fig. 1A and B), the cell proliferation was significantly inhibited (P<0.05) at 2.5 µg/ml concentration. The cell proliferation data for both cell lines shown in Fig. 1A and B represent cumulative data obtained from experiments conducted with SDGE isolated from three different batches of ginger rhizomes. Sixteen replicates were used for each condition tested for each batch of SDGE. Therefore, each data point in Fig. 1 is a mean of 48 separate readings and shows only minor variations in the measurements, clearly demonstrating the highly reproducible effects of the SDGE batches on the proliferation of ECC-1 and Ishikawa cells. Analysis of the data from the proliferation assay demonstrated that at the 72 h timepoint, the IC_50_ of SDGE for both cell lines was approximately 1.25 µg/ml.

### Combined Treatment with SDGE and Radiation or Cisplatin Produces Enhanced Anti-proliferative Effect

Advanced endometrial cancer is treated by using radiation and chemotherapy. We therefore investigated if the combined treatment of the endometrial cancer cells with SDGE and radiation or cisplatin produced an enhanced anti-proliferative effect on ECC-1 and Ishikawa cells. The results of the MTT assay showed that SDGE treatment decreased the proliferation of the two cell lines by 40% and this decrease in proliferation, in the ECC-1 cells, was comparable to that observed in cells that were treated with radiation alone (Fig. 1C and D). In the case of Ishikawa cells, the inhibition of proliferation produced by SDGE was approximately 10% higher than that observed with radiation alone (Fig. 1C). Finally, in the case of both ECC-1 and Ishikawa cells, the combined treatment with SDGE and radiation enhanced inhibitory effect on the proliferation by 23–25% as compared to the cells that were treated only with radiation (Fig. 1C and D).

Next we also tested the combined effect of SDGE and cisplatin on Ishikawa and the ECC-1 cells. MTT assays conducted after 72 h of treatment demonstrated that combined treatment with SDGE and cisplatin decreased the proliferation of the Ishikawa cells by an additional 26% as compared to the cisplatin only treatment (Fig. 1E). In contrast, we did not observe further improvement in the inhibition of proliferation when the ECC-1 cells were treated with a combination of SDGE and cisplatin (Fig. 1F).

These experiments also allowed us to conduct a relative comparison of the cytotoxic effects of SDGE with those of cisplatin. When used as a single agent, Cisplatin (5 µM; 1.5 µg/ml) decreased the proliferation of Ishikawa and ECC-1 cells by 59% and 61%, respectively as compared to the control (Fig. 1E and F). When SDGE (2.5 µg/ml) was used as a single agent, the inhibition of proliferation of Ishikawa and ECC-1 cells was 37% and 42%, respectively, as compared to the controls (Fig. 1E and F). These results suggested that similar to cisplatin, SDGE was also very potent in inhibiting endometrial cancer cell proliferation, providing strong justification for further study of the mechanism by which this botanical extract was producing its anti-cancer effects.

### Inhibition of Endometrial Cancer Cell Proliferation via Apoptosis

**Figure 2:**
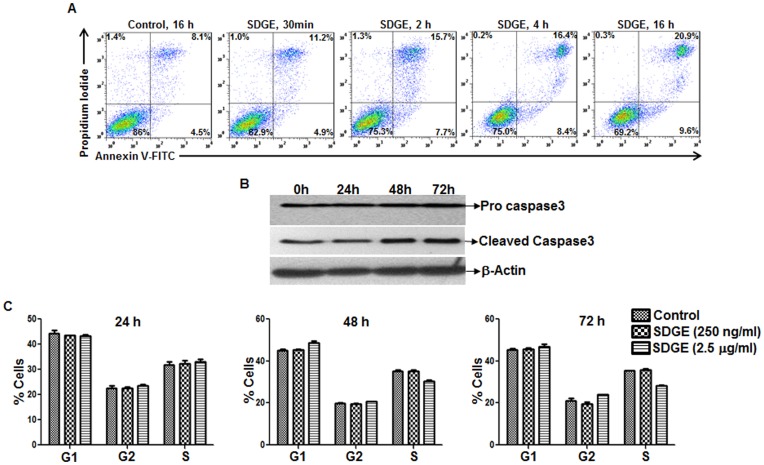
SDGE induces apoptosis in endometrial cancer cells. Ishikawa cells were treated with 250 ng/ml of SDGE for 30 min, 2 h, 4 h, and 16 h. After incubation with SDGE, cell survival was determined by labeling the cells with FITC-conjugated FITC-Annexin V and propidium iodide (A). The cells were analyzed by flow cytometry and cell death and apoptosis were identified as the events that were single positive for FITC-Annexin V (lower right quadrant) or double positive for both FITC-Annexin V and proidium iodide (upper right quadrant). SDGE-induced apoptotic cell death in the endometrial cancer cells was confirmed by detecting cleaved caspase 3 in western blot analysis (B). An increase in cleaved caspase 3 levels was observed when the Ishikawa cells were treated with SDGE (250 ng/ml) for 0, 24, 48, and 72 h. Data in A and B is representative of results obtained in three separate experiments,

The MTT assays indicated that the SDGE was a potent inhibitor of the proliferation of the endometrial cancer cell lines. The decreased proliferation of the endometrial cancer cell lines was a direct result of apoptosis induced in the cells following SDGE treatment. SDGE at concentrations as low as 250 ng/ml caused an increase in the surface binding of FITC-Annexin V and propidium iodide staining as determined by flow cytometry (Fig. 2A). Increase in FITC-Annexin V positive cells was observed as early as 30 min after SDGE was added to the cell cultures (Fig. 2A). Western blot analysis confirmed the increased expression of caspase 3 in the ECC-1 (data not shown) and Ishikawa cells that were treated for 24, 48, and 72 h with 250 ng/ml concentration of SDGE (Fig. 2B). Next we tested if SDGE was also inducing cell cycle arrest in the endometrial cancer cells. Ishikawa cells were therefore treated with 250 ng/ml or 2.5 µg/ml of SDGE for 24, 48, and 72 h. Cells were labeled with propidium iodide and the cell cycle status was monitored by flow cytometry (Fig. 2C). This analysis indicated that SDGE did not induce any major effect on the cell cycle status of the cells. Only marginal decrease was observed in the percentage of cells in the S-phase of the cell cycle. However, this decrease was observed only when the Ishikawa cells were treated with 2.5 µg/ml. Treatment of the cells with 250 ng/ml did not induce any change in the cell cycle status even though this concentration of SDGE induced apoptosis in the cancer cells.

### Chemical Composition of SDGE

**Figure 3:**
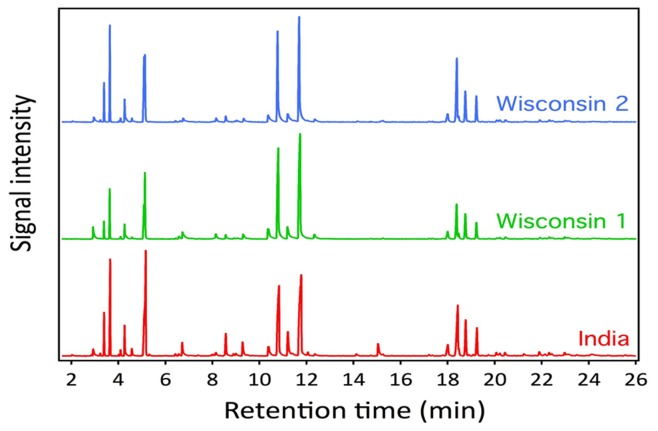
Terpenes are the major components of SDGE identified by GC-MS analysis. Three separate preparations of SDGE, two from the US (termed Wisconsin 1 and 2) and one from India were separated on a non-polar gas chromatography column. The compounds separating on this column were identified by mass spectrometry. Compounds identified through this analysis along with their relative abundances are shown in Table 1.

The potent anti-cancer effects of SDGE prompted us to investigate the potential bioactive components of ginger that likely induce apoptosis in the endometrial cancer cell lines. SDGE isolated from three separate batches of ginger roots were analyzed by GC-MS. The ginger extract was diluted in pentane and the volatile components were separated through a non-polar RTX-5MS column (Fig. 3). The individual peaks eluting from the column were analyzed by electron ionization mass spectrometry. An analysis of the retention indices and mass spectra allowed us to identify a total of 22 compounds in the SDGE (Table 1). The relative percentage of each of the identified component was also determined in this analysis. The data indicated that SDGE isolated from different batches of ginger rhizomes was comparable in its chemical constituents.

**Table 1:** Terpenes identified by GC-MS in SDGE isolated from three separate batches of ginger.

Compound	Relative percentage of the identified terpenes		
	Batch from India	Batch 1 from Wisconsin	Batch 2 from Wisconsin
α-pinene	2.7	1.4	3.2
Camphene	8.1	4.3	8.6
β-pinene	0.4	0.2	0.3
β-myrcene	2.2	1.5	2.3
β-phellandrene	11.0	4.7	9.0
1, 8-cineole	5.6	6.8	6.2
α-terpenoline	0.2	0.1	0.1
2-nonanone	0.2	0.5	0.2
Linalool	1.5	2.2	0.8
Borneol	2.6	0.7	1.0
α-terpineol	1.7	1.0	0.7
Citronellol	1.8	3.1	2.2
Neral	14.8	20.2	15.4
Geraniol	3.6	3.2	3.0
Geranial	21.6	28.1	20.1
bornyl acetate	0.5	1.3	0.8
geranyl acetate	1.8	0.1	0.3
ar-curcurmene	2.0	1.7	2.1
Zingiberene	9.2	6.5	12.3
germancrene-D	0.2	4.8	0.8
β-bisabolene	4.8	4.8	6.0
β-sesquiphellandrene	3.6	2.8	4.6

**Figure 4:**
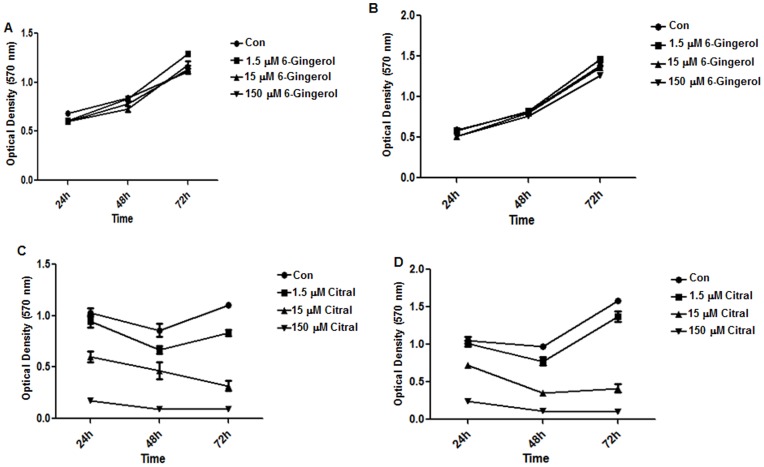
Citral inhibits proliferation of endometrial cancer cells. MTT assays were conducted to determine the effect of 6-gingerol (A and B) and citral (C and D) on the proliferation of Ishikawa and ECC-1 cells. Each data point is a mean of 16 individual readings. Based on this data the IC_50_ of citral was calculated to be 15–25 µM.

The GC-MS analysis also led us to conclude that SDGE does not contain significant amounts of the phenolic compounds, gingerol, shogaol, and paradol that have previously been identified as the anti-cancer agents present in ginger powder and the solvent extracts of ginger rhizomes [20], [24], [25], [36]. This data is supported by our observation that 6-gingerol does not inhibit the proliferation of Ishikawa and ECC-1 cells even when tested at concentrations as high as 150 µM (Fig. 4A and B).

Terpenoids were the major components of SDGE identified in the GC-MS analysis. Neral and geranial are isomers that are collectively referred to as citral. These two compounds constituted approximately 35–45% of the total chemical components identified in our analysis (Table 1). In the *in vitro* assays it was observed that treatment with citral resulted in a significant decrease in the proliferation of Ishikawa and ECC-1 cells. The IC_50_ for citral for both cell lines was between 15–25 µM (Fig. 4C and D). This IC_50_ concentration of citral corresponds to 2.28–3.8 µg/ml. In comparison, the SDGE was effective at an IC_50_ of 1.25 µg/ml. Since only 35–45% of the SDGE is composed of neral plus geranial, the IC_50_ concentration of SDGE corresponds to only a 2.8–3.7 µM concentration of citral. This level of citral is significantly lower than the IC_50_ concentration calculated for this agent in our in vitro proliferation assays (Fig. 4C,D). Therefore, we concluded that in addition to citral, there are other bioactive components that contribute significantly to the anti-cancer effects of SDGE. Hence, we conducted the mechanistic studies listed below using SDGE instead of citral as the bioactive agent.

### SDGE Induces Calcium Flux and Impairs Mitochondrial Membrane Potential

**Figure 5:**
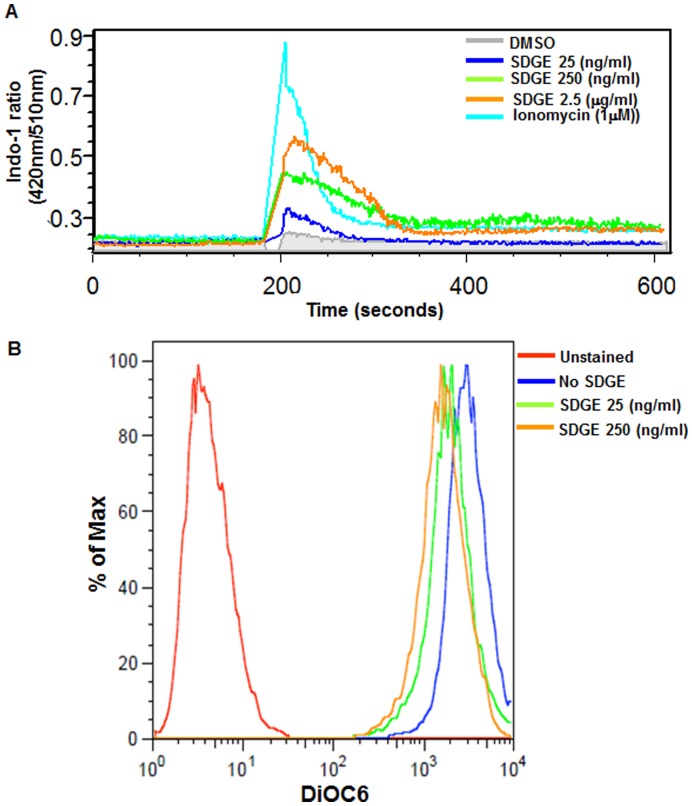
SDGE increases intracellular calcium and decreases the mitochondrial membrane potential of endometrial cancer cells. Effect of SDGE on the intracellular calcium flux was determined by treating Indo-1 loaded Ishikawa cells (A). Immediately after addition of SDGE to the cell suspensions, the Ishikawa cells were flowed through the cytometer and increase in fluorescence was measured to detect calcium flux. Decrease in mitochondrial membrane potential was detected by loading Ishikawa cells with DiOC6 (B). SDGE or vehicle control was added to the cells. After 24 h of treatment, the cells were harvested and incubated with DiOC6 for 15 min. Fluorescence was measured to determine changes in mitochondrial membrane potential.

Treatment of Ishkawa cells with SDGE resulted in a significant influx in intracellular calcium (Fig. 5A). The increase in the intracellular calcium was observed approximately 3 min after addition of SDGE. The time kinetics and the overall profile of the calcium flux were similar to that observed in cells treated with ionomycin. The amplitude of the calcium flux response was dependent on the concentration of SDGE. At the highest concentration of SDGE tested, the maximum calcium flux observed was approximately 60–70% of the response observed with ionomycin (1 µm). Ionomycin induced a sudden increase in intracellular calcium levels which then decreased within a minute but for all concentrations of SDGE tested the rise and subsequent decrease in intracellular calcium was relatively slow (Fig. 5A). Calcium flux decreased 5–6 min after addition of SDGE but did not reach pretreatment levels (Fig. 5A).

The increase in calcium and induction of apoptosis in the SDGE treated ECC-1 and Ishikawa cells suggested a deficit in the mitochondrial function. Measurement of the mitochondrial membrane potential indicated a 2-fold decrease in the Ishikawa cells that were treated for 24 h with 25 ng/ml or 250 ng/ml of SDGE (Fig. 5B).

### SDGE Treatment Results in an Increase in the Bax/Bcl-2 Ratio

**Figure 6:**
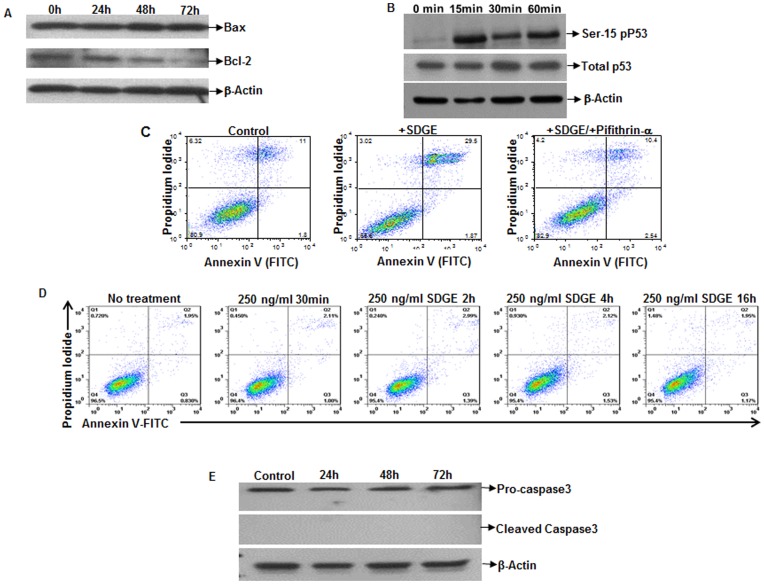
SDGE mediates endometrial cancer cell apoptosis through the activation of p53. After treating Ishikawa cells with SDGE (250 ng/ml) for the designated time points, the cells were harvested and lysates were analyzed by Western blotting for expression of Bcl-2 and Bax (A). Phosphorylation of Ser-15 of p53 was monitored in Ishikawa cells treated with SDGE (250 ng/ml) (B). Ishikawa cells were incubated with SDGE in the presence or absence of pifithrin-α for 18 h. Control Ishikawa cells were not exposed to either SDGE or pifithrin-α. (C) Cells were harvested and apoptosis was measured by flow cytometry after staining with FITC-Annexin V and propidium iodide. Apoptosis in the p53^neg^ SKOV-3 cells after treatment with SDGE (250 ng/ml) was also measured by the FITC-Annexin V assay (D) or by monitoring the levels of cleaved Caspase3 by Western blotting (E) at the designated time intervals.

Bcl-2 is an anti-apoptotic protein that associates with the mitochondrial membrane. Decreases in the mitochondrial membrane potential led us to determine the effect of SDGE (250 ng/ml) on Bcl-2 expression. In both the ECC-1 (data not shown) and the Ishikawa cells (Fig. 6), decreased Bcl-2 expression was observed after 24, 48, and 72 h treatment with SDGE. In contrast, the expression of the pro-apoptotic protein Bax was not altered as a result of the treatment of the endometrial cancer cell lines with 250 ng/ml concentration of SDGE (Fig. 6A). Overall, SDGE treatment resulted in 1.3–2.0-fold increase in the ratio of Bax/Bcl-2 at 24, 48, and 72 h after treatment with 250 ng/ml SDGE.

### SDGE Activates p53

The tumor suppressor p53, a transcriptional factor, plays a key role in regulating cell death and various processes in the cell. The cellular processes regulated by p53 include cell cycle, apoptosis, DNA repair, and senescence. Since SDGE induced apoptosis in endometrial cancer cells, we decided to examine the effect of this extract on p53 activation. SDGE induced a rapid rise in the phosphorylation of p53 protein on Ser-15 (Fig. 6B). This data indicates an activation of the p53 pathway.

Inhibition of p53 by using pifithrin-α, a specific inhibitor [37], resulted in an almost complete reversal of SDGE-induced apoptosis in the cancer cells (Fig. 6C). Approximately, 11% of the control Ishikawa cells that were not treated with SDGE or pifithrin-α were positive for FITC-Annexin V. Treatment with SDGE increased the percentage of FITC-Annexin V positive cells to 39.5%. However, after treatment with pifithrin-α, apoptosis in the SDGE-treated Ishikawa cells was observed in 10% of the population similar to that observed in the vehicle-treated controls (Fig. 6C).

### SDGE does not Induce Apoptosis in p53^neg^ SKOV3 Cells

Previous reports and our own studies have shown that the tumor suppressor gene p53 is not expressed in the ovarian cancer cell line SKOV-3 [38]. Treatment of SKOV-3 cells with SDGE (250 ng/ml) for 30 min, 2, 4, and 16 h did not result in any increase in the apoptosis as determined by FITC-Annexin V binding to the treated cells (Fig. 6D). The lack of apoptosis in the SDGE-treated SKOV-3 cells was further confirmed by the lack of expression of cleaved caspase3 (Fig. 6E).

## Discussion

Ginger either in the form of raw rhizomes, dry powder, or solvent extracts is used in traditional medicine to prevent nausea. Extracts of ginger are also known to possess potent anti-cancer properties. Dry powder of ginger and solvent extract of the rhizome inhibit the proliferation and metastasis of breast, skin, colon, ovary, prostate and other cancer cells [15], [19]. The majority of these studies have suggested phenolic compounds (6-gingerol, shogaol, and others) are responsible for the anti-proliferative effects of ginger [19]. However, careful analysis of the data suggests that these polyphenolic compounds inhibit growth and induce apoptosis of cancer cells at relatively high concentrations of >10 µM [16], [22], [39]. In many studies the phenolic compounds from ginger were shown to inhibit cancer cell proliferation at IC_50_ concentrations of 50–100 µM. The high IC_50_ concentrations of the phenols have likely curtailed additional investigations on the development of ginger-based compounds for the prevention and treatment of ovarian and other solid tumors.

The data from our study demonstrates, however, that there are other bioactive compounds in ginger that exhibit more potent anti-cancer properties. These compounds can be conveniently extracted from ginger by steam distillation. Remarkably, the yields, chemical composition, and the bioactivities of the SDGE isolated from rhizomes procured in Asia and North America are comparable. This consistency in chemical composition and bioactivity is a desirable feature if ingestion of ginger roots or SDGE becomes a recommended approach for prevention and treatment of endometrial cancer.

Our data demonstrates that SDGE treatment results in a rapid increase in the levels of intracellular calcium and in the activation of p53 (Fig. 5 and 6). Furthermore, inhibition of p53 attenuates the ability of SDGE to induce apoptosis in the endometrial cancer cells. SDGE also does not induce apoptosis in the p53^neg^ SKOV-3 cells. These observations support the inference that activation of p53 is the major mechanism by which SDGE inhibits the proliferation of endometrial cancer cells. In this context it is important to note that 6-gingerol and zerumbone, a sesquiterpene found in ginger, also induce cancer cell death by increasing p53 levels and decreasing the Bcl-2/Bax ratio [17], [21], [40]–[42].

Phosphorylation of Ser-15 of p53 interferes with the interaction between this protein and its negative regulator, MDM2 [43]–[45]. As a result, the ubiquitination and subsequent degradation of p53 is inhibited [46]. Activation of p53 decreased the expression of Bcl-2 [47], a result we have also observed in SDGE-treated ECC-1 and Ishikawa cell lines (Fig. 6). Western blot analysis suggests that SDGE decreases the Bcl-2:Bax ratio. Modulation of the Bcl-2/Bax ratio facilitates the formation of the apoptosome [48] thus resulting in apoptotic cell death of the SDGE-treated endometrial cancer cells. Induction of apoptosis by SDGE is supported by the observation that mitochondrial membrane potential is significantly decreased in the endometrial cancer cell lines after SDGE exposure. Thus, activation of p53 is likely a key molecular event that triggers the apoptotic pathway in the endometrial cancer cells.

GC-MS analysis demonstrates that polyphenolic compounds, 6-gingerol, 6-shogaol, and others, are not extracted from ginger rhizomes by our steam distillation protocol. Instead the major components of the SDGE are the terpenoid isomers neral and geranial. The mixture of neral and geranial, citral, was also a potent inhibitor of ECC-1 and Ishikawa proliferation (Fig. 4). However, the IC_50_ of citral in the cell proliferation was approximately 15–25 µM (2.3–3.8 µg/ml). The SDGE, was effective at half maximal concentration of 1.25 µg/ml. These calculations suggest that in addition to citral, other components of the SDGE are also involved in inhibiting the proliferation of the endometrial cancer cells. Our on-going studies will focus on monitoring anti-proliferative properties of the other components of the SDGE. These studies will also investigate if some of the constituent terpenes of SDGE sensitize the cancer cells to apoptosis mediated by neral and geranial.

In conclusion, our results indicate that SDGE or its purified components can serve as affective agents against endometrial cancer. Our demonstration that SDGE treatment may enhance the anti-cancer effects produced by radiation and chemotherapy (Fig. 1) further suggest that this extract of ginger can be used in combination with other therapeutic approaches that are being used for the treatment of endometrial cancer. It is also possible that ingestion of SDGE may serve as an important chemopreventive strategy for endometrial cancer. The fact that ginger is safely ingested as a dietary component gives us confidence that administration of SDGE under medically controlled conditions should lead to low toxicity while decreasing endometrial tumor growth and metastasis.
